# Blocking myostatin: muscle mass equals muscle strength?

**DOI:** 10.1002/jcsm.12647

**Published:** 2020-12-19

**Authors:** Markus S. Anker, Stephan von Haehling, Jochen Springer

**Affiliations:** ^1^ Berlin Institute of Health Center for Regenerative Therapies (BCRT) Charité Universitätsmedizin Berlin Berlin Germany; ^2^ German Centre for Cardiovascular Research (DZHK) partner site Berlin Charité Universitätsmedizin Berlin Berlin Germany; ^3^ Division of Cardiology and Metabolism, Department of Cardiology (CVK) Charité University Medicine Berlin Berlin Germany; ^4^ Department of Cardiology (CBF) Charité University Medicine Berlin Berlin Germany; ^5^ Department of Cardiology and Pneumology University of Göttingen Medical Center and German Center for Cardiovascular Research (DZHK) partner site Göttingen Göttingen Germany

Myostatin also known as growth differentiation factor 8 (GDF‐8) has been of major interest in the cachexia/sarcopenia/muscle wasting community since its discovery by McPherron *et al*. in 1997.^1^ Naturally occurring mutations leading to a faulty non‐functional myostatin have been described in Belgian Blue and Piedmontese cattle as well as in whippets which show a muscle mass increase of approximately 40%.^2^, ^3^ Disturbed myostatin signalling has also been confirmed in two children, one with a homozygous mutation in the myostatin gene^4^ and one with a mutation in the activin receptor type‐2B (ActRIIB) both leading to a vast increase in muscle mass

Muscle wasting is frequently observed in the elderly population and patients with chronic diseases, in up to 50% of patients.^5^, ^6^ In several disease models of muscle wasting, an upregulation of myostatin has been shown including cancer cachexia,^7^, ^8^, ^9^, ^10^ chemotherapy,^11^ kidney failure,^12^, ^13^, ^14^, ^15^ heart failure,^6^ spinal muscular atrophy,^16^ vitamin D deficiency in infantile nephropathic cystinosis,^17^ glucocorticoids,^14^, ^18^ and oculopharyngeal muscular dystrophy (OPMD).^19^ This shows that myostatin signalling in humans is indeed a valid target for therapeutic interventions in various muscle wasting conditions. However, there seem to be a high number of translation failures in the development of myostatin targeting therapeutics.

Myostatin‐targeting antibodies and soluble ActRIIB to block atrophic signalling in skeletal muscle have been studied extensively in animal models and human trials with varying success. In a progeric mouse model, the soluble ActRIIB‐Fc (ACE‐031, Acceleron Pharma) improved muscle mass and delayed morbidity.^20^ In a Duchenne muscular dystrophy study, the primary endpoint of safety was met, and the study showed a trend for maintenance of the 6 min walk test, lean body mass, and bone mineral density versus placebo without reaching statistical significance.^21^ Atara Biotherapeutics PINTA745 showed good efficacy in a stroke mouse model, in which it attenuated loss of body weight and improved body weight recovery after cerebral ischaemia. More importantly, it also improved muscle strength and motor function.^22^ However, it was unsuccessful in a phase II clinical trial in renal failure, as it did not meet its primary endpoint of lean mass increase (https://www.thepharmaletter.com/article/atara‐halts‐development‐of‐pinta‐745). A second monoclonal antibody—ATA 842—by Atara Biotherapeutics increased muscle mass and strength, as well as insulin sensitivity in old mice over a period of 4 weeks^23^ but seems to remain in the preclinical stage. Pfizer's domagrozumab (B5161002) monoclonal antibody shared the fate of showing good preclinical efficacy and failing in a phase II safety and efficacy study, where the primary endpoint of change from baseline in 4 Stair Climb following 1 year of treatment with domagrozumab as compared to placebo in patients with Duchenne's muscular dystrophy (DMD) (https://www.pfizer.com/news/press‐release/press‐release‐detail/pfizer_terminates_domagrozumab_pf_06252616_clinical_studies_for_the_treatment_of_duchenne_muscular_dystrophy). However, in the mouse DMD mdx model, the mouse analogue of domagrozumab—mRK35—significantly increased body weight, muscle weights, grip strength, and *ex vivo* force production in the extensor digitorum longus (EDL) muscle.^24^ Domagrozumab itself dose‐dependently increased lean mass and muscle volume in non‐human primates.^24^ Eli Lilly's LY2495655 myostatin blocking antibody did not show effects on overall survival or progression‐free survival and hence the trial was terminated due to imbalance in death rates between the treatment arms.^25^ However, in a subgroup of patients with a weight loss of less than 5%, LY2495655 show beneficial effects on muscle mass and function.^25^ A human dual‐specific anti‐ActRIIA/ActRIIB antibody [bimagrumab (BYM338)] has been developed by Novartis, which not only blocks myostatin binding but also that of activin A. The effects of bimagrumab on muscle mass and strength were greater than blocking the ActRIIA or ActRIIB alone in naïve SCID mice over a period of 4 weeks.^26^ In a phase II sarcopenia trial, bimagrumab treatment over 16 weeks increased muscle mass and strength in older adults and improved mobility in those with slow walking speed.^27^ It also improves body composition and insulin sensitivity in insulin‐resistant individuals^28^ and accelerated recovery of muscle mass while reducing intramuscular fat in disuse atrophy induced by an immobilizing cast.^29^ In a small sporadic inclusion body myositis trial bimagrumab increased lean body mass and thigh muscle volume resulting in an improved 6 min walking distance.^30^

In this issue of the *Journal of Cachexia, Sarcopenia and Muscle*, Rooks *et al*.^29^ describe the safety profile, pharmacokinetics, and pharmacodynamics of the human monoclonal antibody bimagrumab, which blocks the activin type II receptors, in healthy older and obese adults. Bimagrumab was safe and well tolerated, and the pharmacokinetics were similar in both studies. A rapid increase of lean body mass and thigh muscle volume was observed, while fat mass decreased. In the high dose group (30 mg/kg, single iv. infusion), the increase of lean mass was maintained over 4 weeks, while the 3 mg/kg dose did not. Unfortunately, no improvement of muscle strength/function was observed, but this may be due to short study duration and single dosing of bimagrumab.

In general, there seems to be no direct relationship between muscle mass and strength,^31^ making the development of myostatin pathway targeting therapeutics very challenging at best.

## Conflict of interest

The authors have no conflict of interest regarding the subject of this editorial.
